# Carbon Based Electrodes Modified with Horseradish Peroxidase Immobilized in Conducting Polymers for Acetaminophen Analysis

**DOI:** 10.3390/s130404841

**Published:** 2013-04-11

**Authors:** Mihaela Tertis, Anca Florea, Robert Sandulescu, Cecilia Cristea

**Affiliations:** Analytical Chemistry Department, Faculty of Pharmacy, Iuliu Haţieganu University of Medicine and Pharmacy, 4 Pasteur St., Cluj-Napoca 400349, Romania; E-Mails: mtertis@chem.ubbcluj.ro (M.T.); Florea.Anca@umfcluj.ro (F.A.); rsandulescu@umfcluj.ro (R.S.)

**Keywords:** horseradish peroxidase, single and multi-wall carbon nanotubes, carbon based electrodes, conducting polymer, nanocomposites, acetaminophen

## Abstract

The development and optimization of new biosensors with horseradish peroxidase immobilized in carbon nanotubes-polyethyleneimine or polypyrrole nanocomposite film at the surface of two types of transducer is described. The amperometric detection of acetaminophen was carried out at −0.2 V *versus* Ag/AgCl using carbon based-screen printed electrodes (SPEs) and glassy carbon electrodes (GCEs) as transducers. The electroanalytical parameters of the biosensors are highly dependent on their configuration and on the dimensions of the carbon nanotubes. The best limit of detection obtained for acetaminophen was 1.36 ± 0.013 μM and the linear range 9.99–79.01 μM for the HRP-SWCNT/PEI in GCE configuration. The biosensors were successfully applied for the detection of acetaminophen in several drug formulations.

## 1. Introduction

Carbon nanotubes (CNTs) rapidly became in the last decade the building blocks of choice in various nanotechnology applications, due to their electronic transport properties, high mechanical strength, chemical properties and increased thermal conductivity [1]. CNTs can act as semiconductors, insulators or metals, depending on their structure, and these features are exploited in the construction of electrochemical sensors. CNTs can be integrated in a variety of configurations in order to improve the electrochemical detection.

Amperometric biosensors based on CNTs continue to be reported in the literature. While many of these electrodes exhibit promising results compared to other carbon-based electrodes, there are few studies comparing different varieties of CNT-based electrodes [2]. Consequently, CNTs were integrated in conductive polymer matrix in order to create a nanocomposite material. Single and multiwall CNTs (SWCNTs and MWCNTs) were used to modify the surface of a conventional glassy carbon electrode and also to modify and improve the electrochemical behaviour of carbon based screen-printed electrodes [3].

A crucial step in the development of biosensors is the fast electron-transfer between the active site of the enzyme and the electrochemical transducer. A number of methods have been used to immobilize the redox enzyme on the electrode surface and in the same time to preserve the enzymatic activity [4,5]. Literature data indicate that CNTs promote electron-transfer reactions at low overpotentials in the presence of several enzymes like glucose oxidase [6,7], glucose dehydrogenase [8], and horseradish peroxidase [9]. The incorporation of SWCNTs and MWCNTs into layer by layer (LBL) films containing enzymes [10] enhances the electron diffusion through the films and the electrochemical surface of the electrode, thus increasing the sensor response. In the same time, the adsorption of enzymes directly on the CNTs reduces the distance between the active site of enzyme and the surface of the electrode [11,12]. CNTs provide also homogeneous porous composite films [13] and are considered ideal conducting nanowires to establish efficient direct electron transfer between the active site of an enzyme and the electrode surface [7].

Another important consideration is the enzyme entrapment method. Conducting polymers (CPs), due to their structure, provide versatile electronic properties (metallic to insulator), low ionization potential and high electron affinity, offering an easy to use method for enzyme immobilization. However, most of these properties depend on the synthesis procedure used as well as on the nature of the polymer itself [14].

A popular strategy for CNT-based biosensors construction is to immobilize CNTs onto electrodes using polymer coatings. Polypyrrole (PPy) has been the focus of several recent studies during the last twenty years, because it has high conductivity and good environmental stability and can be overoxidized to create an electrically insulating layer [15–18]. Besides polypyrrole, another interesting polymer is polyethyleneimine (PEI). PEI is a cationic polymer used for the entrapment of several molecules in various biosensors configurations [19]. During the development of the novel biosensors for acetaminophen detection, the use of PEI offered the advantage that the retention of the biomolecule at the electrode surface was made without stressing it with a supplementary electropolymerisation process [20,21].

N-acetyl-*p*-aminophenol (4-acetamidophenol, known as acetaminophen—APAP) is an acylated aromatic amide that has been used as an analgesic for over 30 years, being a very effective treatment for the relief of pain and fever in adults and more often in children. Recent studies have shown that it is associated however to hepatic toxicity and renal failure at overdoses after long term use [22].

Screen printed electrodes (SPEs) are miniaturized devices, based on different layers of inks printed on an inert substrate and have been developed for many innovative applications in analytical chemistry. As reported in the literature, the use of SPEs offers a very simple and flexible design and operation in analytical determinations, in accordance with the requirements of a decentralized assay [23]. Recently, a great interest in SPEs has been demonstrated, especially in the development of rapid analytical analyses and the fabrication of biosensors. Moreover, regarding their simplicity, they are simply mass-produced at low cost, having at the same time superior characteristics in comparison with the currently used electrodes.

Horseradish peroxidase (HRP) has been a powerful tool in biomedical and pharmaceutical analysis. The wide use of HRP is due to its commercial availability in a relatively pure form at reasonable low prices, stability, and its high turnover on a variety of substrates. In this study HRP was immobilized onto the transducer surface (SPEs or GCE) by entrapment into polymer films (PEI and Ppy) doped with SWCNT and MWCNT.

**Figure 1.**
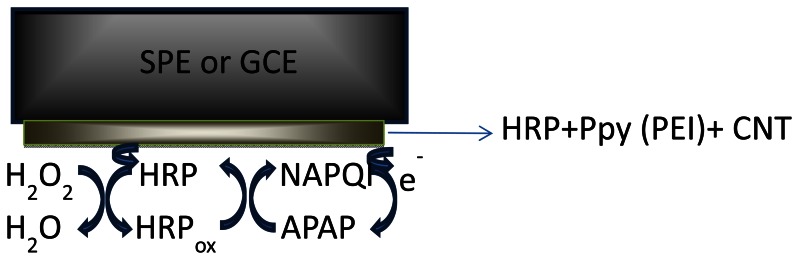
The biosensor configuration.

**Scheme 1.**
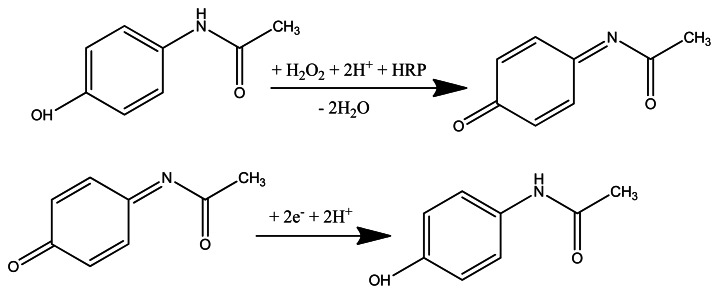
Enzymatic and electrochemical transformations of acetaminophen in aqueous media.

The obtained configurations (summarized in Figure 1), were used to monitor the signal produced by the electrochemical reduction of the enzymatically generated electroactive oxidized species of acetaminophen (N-acetylbenzoquinoneimine—NAPQI) in the presence of hydrogen peroxide by amperometry, cyclic voltammetry (CV) and differential pulse voltammetry (DPV) [24] (Scheme 1).

A comparative study between different types of CNTs for doping the polymeric films was performed. The biosensors were applied to the assay of acetaminophen in various drug formulations (mono and bicomponent) by the standard addition method.

## 2. Experimental Section

### 2.1. Chemicals and Equipment

Single and multiwall carbon nanotubes were purchased from NanoLab Inc. (Waltham, MA, USA). Horseradish peroxidase enzyme (1.11.1.7 type II, 180 U/mg), hydrogen peroxide 30%, monosodium phosphate and the disodium phosphate were purchased from Sigma-Aldrich (St. Louis, MO, USA). Polyethyleneimine (MW 60000) (PEI) from Sigma-Aldrich (St. Louis, MO, USA) was used without purification. The stock 10^−2^ M solutions of acetaminophen (Merck, Whitehouse Station, NJ, USA) were dissolved in phosphate buffer and kept in the refrigerator. All reagents were of analytical grade, and used as received. Carbon based screen-printed electrodes (SPEs) were purchased from Dropsens (Llanera, Spain) and consisted in graphite working and auxiliary electrodes and a silver pseudo-reference electrode. The glassy carbon electrodes (GCEs) used as working electrode in the conventional three electrode cell were purchased from BAS Inc. (West Lafayette, IN, USA) and eDAQ (Denistone East, Australia) and were carefully washed with demineralized water and polished with diamond paste (BAS Inc., West Lafayette, IN, USA). In order to obtain a better dispersion, 20 mg of SWCNTs with 1.5 nm diameter and 1–5 μm length from NanoLab Inc. were sonicated in 300 mL tetrahydrofuran (THF) for 2 hours. The solvent was then evaporated. The obtained solid was then diluted with dimethylformamide (DMF) and sonicated again for 2 h. After sedimentation of undispersed nanotubes, the supernatant was filtered and washed with THF. The solid was not allowed to dry during filtration, but transferred in 5 mL THF and mechanically stirred until complete solvent evaporation to avoid SWCNTs aggregation. SWCNTs were dried overnight at 70 °C. A homogeneous SWCNT dispersion can be obtained after 15 min of sonication before every use [25,26].

### 2.2. Nanocomposite Material Preparations and HRP Immobilization

One mg of each type of MWCNT was suspended in PEI solution, (1 mL, 1 mg·mL^−1^ prepared in 50:50 v/v ethanol/water) or in pyrrole solution (5 mM) prepared in LiClO_4_ (0.1 M) followed by 15 min sonication. Prior to enzyme coating, the glassy carbon working electrodes (diameter 1 mm and 4 mm) were polished with diamond paste on felt pads and then washed with ethanol, acetone and deionized water. Equal amounts of the MWCNT/PEI and MWCNT/Ppy suspension and a 0.3 mg·mL^−1^ or 0.6 mg·mL^−1^ HRP solution in phosphate buffer solution (PBS) (pH 7.4; 0.1 M) were mixed with vortex for 5 min. A small aliquot (10 or 20 μL) of this mixture was deposited on the working electrode (GCE and graphite based SPEs) and dried at room for 2 h. The electropolymerization of pyrrole was carried out by cycling the potential between 0 and +0.8 V, at 100 mV·s^−1^ scan rate for 20 cycles, in LiClO_4_ (0.1 M), followed by an overoxidation by cycling between 0 and +1.2 V, 100 mV·s^−1^ scan rate for 5 cycles, in LiClO_4_ (0.1 M). The SWCNTs GCE and SPE working surfaces were modified by depositing successive layers of 10 μL of the THF dispersion and drying the solvent of each layer. 15 layers of SWCNTs were formed on the surface of the electrode in this manner (layer-by-layer construction [26]). The electrodes were then rinsed with THF and water to remove the unattached SWCNTs and residues of the organic solvent. Two drops of enzyme containing solutions of different concentrations (in 1 mg·mL^−1^ PEI or 5 mM Py solution) were applied onto the electrode, left to air dry, then the electropolymerization of monomer was performed by cyclic voltammetry (in the case of the Py modified electrode). The biosensors were stored at 4 °C, in a semidry state, leaving a small droplet of buffer solution on top, to maintain a humid environment. Figure 1 shows the schematic representation of the obtained biosensors.

### 2.3. Electrochemical Methods

The experiments were achieved by using an AUTOLAB PGSTAT 30 (Ecochemie, Utrecht, The Netherlands) equipped with GPES and FRA2 software. All the experiments were performed in PBS (pH 7.4; 0.1 M) at room temperature (25 °C). The pH of the solution was controlled by a ChemCadet pH-meter (Cole Parmer, Chicago, IL, USA). The cyclic voltammetry determinations were recorded in the presence of hydrogen peroxide 0.2 mM, with 100 mV·s^−1^ scan rate, in a conventional three electrodes setup and by using the modified SPEs. During amperometric experiments the biosensor potential was kept at −0.2 V *vs.* Ag/AgCl under continuous stirring conditions. The working potential was imposed and the background current was allowed to arrive at a steady state value. Different amounts of acetaminophen standard solution were added, every 100 seconds, into the stirred electrochemical cell and the current was recorded as a function of time. The DPV method was also used and the optimised parameters are: modulation time 0.05 s, step potential 0.01 V, modulation amplitude 0.07 V between a potential of −0.6 and +2 V.

### 2.4. UV Spectrophotometry Determinations

The spectrophotometry determinations were performed on SPECORD 250 PLUS spectrophotometer (Analitic Jena, Jena Thuringia, Germany) equipped with WinAspect software. Acetaminophen was determined by spectrophotometry in UV at 243 nm, the method recommended by the 10th edition of the Romanian Pharmacopoeia. A quantity of acetaminophen (0.1 g) was dissolved in 0.05 M H_2_SO_4_ (50 mL) and brought to mark with the same solvent in a 100 mL volumetric flask. One mL of this solution was transferred in a 100 mL volumetric flask and brought to the mark with 0.05 M H_2_SO_4_. The specific absorbance $\left(E_{1cm}^{1\%}\right)$ of 645 for acetaminophen solution was recorded at 243 nm [27].

### 2.5. Procedure for Real Sample Analysis

Ten tablets of each analyzed pharmaceutical formulation were accurately weighed and finely powdered in a mortar. An adequate amount of the powdered tablets were dissolved in 75 mL PBS (pH 7.4; 0.1 M). The suspension was stirred for 30 min., filtered and then transferred to a 100 mL volumetric flask and completed to volume with PBS (pH 7.4; 0.1 M) (predicted acetaminophen concentration was 10^−2^ M). The concentration of APAP was calculated by using the standard addition method. The standard addition method was also used for analyzing the pharmaceutical samples. To test the interference between APAP and caffeine with codeine phosphate in bicomponent pharmaceutical formulations the DPV method was used to prove the separation of the oxidation peaks. For this purpose an adequate amount of the powdered tablets (of Panadol Extra® containing APAP and caffeine and Paradoren® containing APAP and codeine phosphate as active species) were dissolved in 75 mL PBS (pH 7.4; 0.1 M). The suspension was stirred for 30 min., filtered and then transferred to a 100 mL calibrated flask and completed to volume with PBS (pH 7.4; 0.1 M) (predicted caffeine/codeine phosphate concentration was 10^−3^ M).

The amperometric experiments were performed under continuous stirring condition. Different amounts of acetaminophen solution (obtained from different pharmaceutical formulation) were added, every 100 seconds, into the stirred electrochemical cell and the current was recorded as a function of time. In the case of spectrophotometry determinations performed on real samples (solutions containing acetaminophen obtained from pharmaceuticals), a quantity of powder that should contain 0.1 g acetaminophen was stirred for 30 minutes in 50 mL of 0.05 M H_2_SO_4_, brought to mark with the same solvent in a 100 mL volumetric flask then filtered. One mL of this solution is brought to mark with 0.05 M H_2_SO_4_ in a 100 mL volumetric flask, and then the absorbance at 243 nm was determined.

## 3. Results and Discussion

### 3.1. CNTs Conducting Polymers Nanocomposites Modified with HRP Electrochemical Behaviour

Five types of CNTs were tested in order to establish the best electrochemical behavior for the development of the biosensor (Table 1).

**Figure 2.**
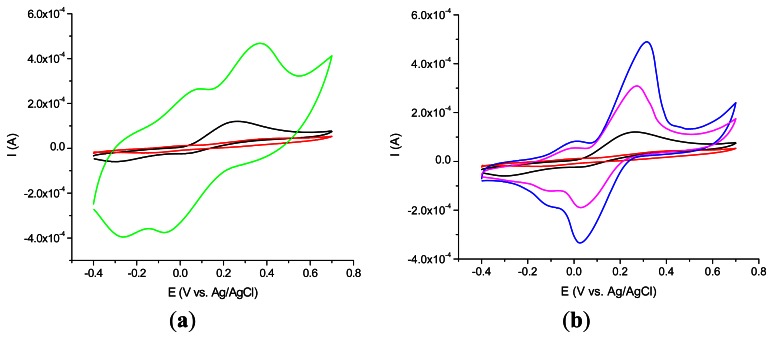
The electrochemical behavior of 10−4 M acetaminophen in PBS (pH 7.4; 0.1 M) at: (**a**) SWCNT/PEI (green) and (**b**) MWCNT (type 1)/PEI (blue); MWCNT (type 3)/ PEI (magenta); compared with GCE simple (black) and GCE modified with PEI (red).

The electrochemical behavior strongly depends on the diameter and the length of each type of CNT tested as shown in Figure 2.

**Figure 3.**
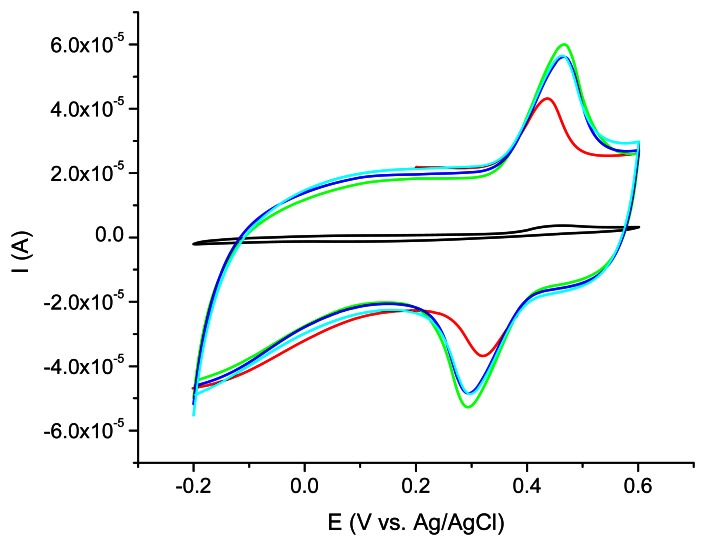
Cyclic voltammetry of nanocomposite MWCNT (type 1)/Ppy film permeability on SPE tested in the presence of 10^−4^ M acetaminophen in 0.1 M LiClO4, 10 cycles (red), 20 cycles (green), 40 cycles (blue), 60 cycles (cyan) compared with unmodified GCE 5 cycles (black).

The best acetaminophen electrochemical oxidation results were obtained for MWCNT (types 1 and 3) in cyclic voltammetry. The electrochemical behavior of the nanocomposite CNT/Ppy was investigated by cyclic voltammetry at glassy carbon electrode in water solution containing 0.1 M LiClO_4_ as supporting electrolyte. The repetitive scanning of the electrode potential between −0.2 and 0.8 V induces the appearance and the growth of a quasi-reversible peak system, located around 0.4 V (Figure 3). This evolution indicates the formation and growth of a Ppy film on the glassy carbon surface [16]. Raising the number of cycles over 20 is not correlated with a better electrochemical signal showing that after this number of cycles a thicker layer is formed at the electrode surface. For further investigations the 20 cycles procedure was chosen.

To test the permeability of the film formed at the surface of the electrodes and also the electrocatalytic effect of CNT entrapped in the polymeric films the electrochemical behavior of the acetaminophen was investigated.

**Figure 4.**
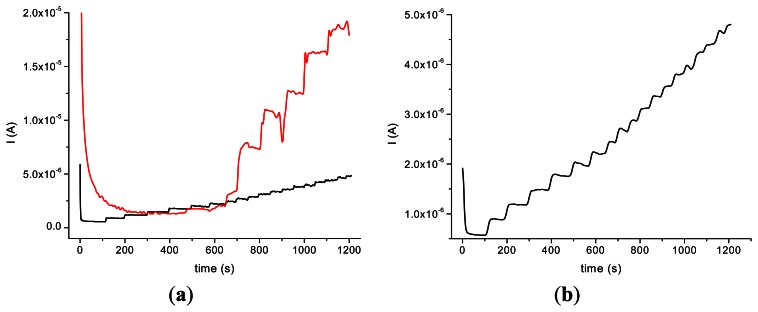
Acetaminophen amperograms at GCEs modified with HRP/MWCNT (type 1) immobilized in Ppy (black) and PEI (red) nanocomposite films (**a**). Detailed version of acetaminophen amperogram at GCEs modified with HRP/MWCNT (type 1) immobilized in Ppy (**b**); (HRP concentration: 0.3 mg·mL^−1^ PEI 1 mg·mL^−1^), after the addition of constant volumes (20 μL) of 10^−2^ M acetaminophen solution in PBS (pH 7.4; 0.1 M) in the presence of 0.2 mM H_2_O_2_.

The incorporation of the HRP in the nanocomposite allowed us to perform amperometric studies with the obtained biosensors in the presence of H_2_O_2_ and by successive additions of acetaminophen. The amperometric experiments revealed a better response in the case of GCE modified with MWCNT (type 1)/Ppy and a good stability of the HRP/MWCNT/Ppy nanocomposite compared with the HRP/MWCNT/PEI (Figure 4).

It could be assumed that the partial water solubility of PEI and the low dispersion of MWCNT are responsible for this behavior. The response time is longer in the case of HRP/MWCNT/PEI/GCE than the one observed on HRP/MWCNT/Ppy/GCE (500 s compared to 100 s for the latter) probably due to the loss of nanocomposite in bulk solution. In spite of the fact that the current response was bigger in the case of HRP/MWCNT/PEI/GCE compared with the sensors with Ppy, the poor reproducibility and the rapid loss of the signal made difficult the electroanalytical characterisation of the biosensor.

The results obtained on GCE modified with HRP/MWCNT/PEI tested in the presence of H_2_O_2_ 0.2 mM and by adding constant volumes (20 μL) of 10^−2^ M acetaminophen allowed us to obtain a calibration plot with the parameters: Y = −0.0118*x* − 1.725 × 10^−6^; R^2^ = 0.928 (seven points). A better dispersion of the CNTs into the polymeric films was suggested by the poor reproducibility in the case of the nanocomposite with MWCNT and PEI. In the case of GCE modified with HRP/MWCNT/Ppy the calibration data are: Y = −0.0164 *x* − 1 × 10^−6^; R^2^ = 0.984 (seven points).

In order to improve the reproducibility, sensitivity and the mechanical stability of the biosensor the layer-by-layer method was used instead of direct deposition of a small aliquot of HRP/SWCNT/Ppy suspension on the surface of the working electrode (GCE diameter 1 and 4 mm, respectively, and SPE).

Amperometric measurements were also performed in order to compare MWCNTs and SWCNTs response in the nanocomposite. It was observed that the SWCNT/Ppy and SWCNT/PEI nanocomposites behaved better than those obtained with MWCNTs. Compared to MWCNTs, it could be assumed that the simple structure of the SWCNT is more suitable for the electron transfer between the HRP and the electrode modified with a conductive polymer.

The results obtained on SP modified electrodes are inconclusive and present poor reproducibility, probably due to the use of THF for SWCNT suspension preparation, which could degrade the electrode surface. For this reason further experiments were performed on GC modified electrodes.

### 3.2. Optimization of the Bisosensors

Different numbers of layers were tested in order to optimize the response of the biosensors. The best results were obtained for 15 cycles. By decreasing the numbers of cycles the stability of the biosensors is lower and the sensitivity also decreases. Increasing the number of layers didn't improve the sensitivity of the sensors.

The influence of the enzyme loading in the nanocomposite was also investigated by varying the quantity of the entrapped enzyme (0.3 and 0.6 mg·mL^−1^ of HRP polymer solution). A two-fold increase in the enzyme concentration did not double the sensitivity, so for testing the biosensor on real samples the concentration of 0.3 mg·mL^−1^ of HRP in the polymer solution was used.

**Figure 5.**
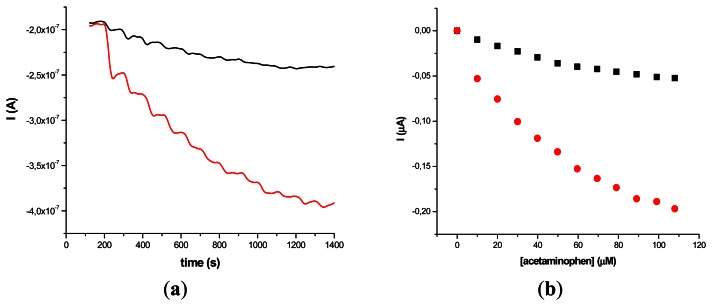
Amperometric response (**a**) and calibration plot (**b**) for 10^−2^ M acetaminophen at GCE (Φ 1 mm) modified with HRP/SWCNT/PEI film; (HRP concentration: 0.3 mg·mL^−1^ (black) and 0.6 mg·mL^−1^ in PEI 1 mg·mL^−1^ (red)), after the addition of constant volumes (5 μL) of 10^−2^ M acetaminophen solution in PBS (pH 7.4; 0.1 M) in the presence of 0.2 mM H_2_O_2_.

**Figure 6.**
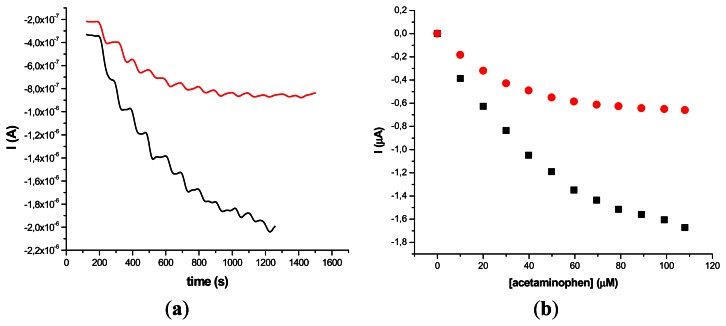
Amperometric response (**a**) and calibration plot (**b**) acetaminophen at GCE (Φ 4 mm) modified with HRP/SWCNT/PEI film; (HRP concentration: 0.3 mg·mL^−1^ (black) and 0.6 mg·mL^−1^ in PEI 1 mg·mL^−1^ (red)), after the addition of constant volumes (10 μL) of 10^−2^ M acetaminophen solution in PBS (pH 7.4; 0.1 M) in the presence of 0.2 mM H_2_O_2_.

The results obtained on two different types of HRP/SWCNT/PEI modified GCE after the addition of constant volumes (5 μL) of 10^−2^ M acetaminophen solution in PBS (pH 7.4; 0.1 M) and 0.2 mM H_2_O_2_ are presented in Figures 5 and 6, where amperometry was used for the electroanalytical characterization of the biosensors. The applied working potential was −0.2 V *vs.* Ag/AgCl.

Comparing the two types of GC modified transducers (GCE provided by BAS and eDAQ) different behaviour of the elaborated biosensors was observed—for the small diameter electrodes the electron transfer is fast, and the best amperometric response is obtained for a concentration of 0.6 mg·mL^−1^ HRP (Figure 5a). In the case of GCE (Φ 4 mm) doubling the HRP concentration does not improve the biosensor response compared with 0.3 mg·mL^−1^ concentration (Figure 6a). For further experiments the following electrodes were used: 0.3 mg·mL^−1^ HRP/SWCNT/PEI/GCE (Φ 4 mm) and 0.6 mg·mL^−1^ HRP/SWCNT/PEI/GCE (Φ 1 mm).

The time required to reach 95% of the maximum steady-state current was 18 s in the case of 0.3 mg·mL^−1^ HRP/SWCNT/PEI/GCE and 22 s in the case of 0.6 mg·mL^−1^ HRP/SWCNT/PEI/GCE. The response was linear in the range from 4.08 μM to 79.01 μM for 0.6 mg·mL^−1^ HRP/SWCNT/PEI/GCE (Φ 1 mm) and from 24.27 μM to 118.06 μM for 0.48 mg·mL^−1^ HRP/SWCNT/Ppy/SPE configuration, respectively. The apparent Michaelis-Menten constant was calculated using the Michaelis-Menten equation:

$$i=i_{\text{max}}-K_{M}^{\text{app}}\;\left(\overset{i}{\;}/\underset{C}{\;}\right)$$

where i is the steady-state catalytic current, *i_max_* the maximum current measured under saturated substrate conditions, C referred to the acetaminophen and stands for the apparent Michaelis-Menten constant of the system.

The value obtained for the apparent Michaelis-Menten constant was 32.23 mM for 0.3 mg·mL−1 HRP/SWCNT/PEI and 17.89 mM in the case of 0.6 mg·mL^−1^ HRP/SWCNT/PEI, both deposited on 4 mm Φ GCE. The results show that the biosensor possesses high biological affinity to acetaminophen and are in agreement with the earlier reports [28].

A comparison between the analytical parameters obtained as a function of the configurations of the biosensors is presented in Table 2. The detection limit for five configurations was calculated for a signal to noise ratio of 3. The lowest limit of detection was obtained for the 0.3 mg·mL^−1^ HRP/SWCNT/PEI modified GCE (Φ 1 mm).

### 3.3. Real Sample Analysis

The obtained biosensors were applied for the assay of acetaminophen in a couple of commercial drug formulations: Panadol^®^ (GlaxoSmithKline) and Panadol Extra^®^ (containing acetaminophen and caffeine, GlaxoSmithKline). The standard addition method was used by adding approximately equal aliquots of acetaminophen (10 μM), from the Panadol^®^ and Panadol Extra^®^ formulation and from a standard 10^−2^ M acetaminophen solution in 0.1 M phosphate buffer (pH 7.4) in the presence of 0.2 mM H_2_O_2_. The results obtained are consistent with those reported by the manufacturer (500 mg/tablet), see Table 3.

As it can be observed in Table 3, the amount of active species found in the drug samples is in good accordance with the labelled amounts. The recovery rates of the active substance in pharmaceuticals containing acetaminophen only are between 99.1% and 100.4%, interferences with all other formulation excipients being negligible.

In the case of pharmaceuticals containing two active compounds (Panadol Extra^®^ containing acetaminophen and caffeine, respectively, and Paradoren^®^ 30/500 containing acetaminophen and codeine phosphate) the recovery rates are lower, between 90.74% and 91.02%. Similar recovery data were obtained in our previous paper [29] where some pharmaceutical formulation containing acetaminophen alone and in various combination with some alkaloids (caffeine, codeine phosphate *etc.*), were determined on electrochemically activated glassy carbon electrodes. It could be assumed that the presence of other active compounds affect the enzymatic activity of the HRP immobilized in the nanocomposite CNT-conductive polymer in the amperometric experiments. Taking into account that the experimental time in amperometry is long, the same electrodes were tested also by DPV where the contact time between the enzymes immobilized at the surface of the electrode and the sample is shorter.

For all the investigated pharmaceuticals the oxidation potential of acetaminophen was found at the same value (E = +0.65 V *vs.* Ag/AgCl). Figure 7 presents the DPVs obtained for Panadol Extra^®^, with 65 mg caffeine and 500 mg acetaminophen per tablet. From the data obtained on the bicomponent solutions (10^−3^ M caffeine) it can be observed that the oxidation potentials are well separated (ΔE about 1 V) and this clearly allows the simultaneous determination of the compounds without further separations.

In order to compare the electroanalytical results, acetaminophen solutions (standard and obtained with the investigated pharmaceuticals) were analysed by spectrophotometry at 243 nm, the official analytical method recommended by the Romanian Pharmacopoeia (Table 4). The obtained results are in good agreement with those obtained by electrochemical method using the new biosensors, both for pharmaceutical products containing acetaminophen only and those with two active compounds. Regarding the declared content of active substance in tablets, Romanian Pharmacopoeia requests a deviation of ±5% [26].

The reproducibility and stability are the two important parameters for the evaluation of the performance of the sensor. The reproducibility of the biosensors was examined at 10 μM acetaminophen solution. The relative standard deviation was between 1.31% and 2.56% for HRP/SWCNT/PEI at GCE (Φ 4 mm) configuration and 1.78% for HRP/SWCNT/PEI at graphite based SPE, respectively (n = 5). Comparing the modified SPEs with the modified GCEs it could be underlined that the RSD and the recovery are better in the case of GCE as working electrode. This could be explained by the roughness of the SPE that lead to a non uniform distribution of the SWCNT and HRP at their surfaces. The results are in agreement with other previous results obtained on Zr alkoxide/HRP/PEI/SPEs and Zr alkoxide/HRP/PEI/GCEs [19,20].

In order to demonstrate the stability, the response of the acetaminophen was measured every day for 10 days. All electrodes, when not in use, were stored under semi-dry conditions and were stable for at least 10 days. 65% of the original biosensor response remained after 10 days. The decrease in current response may be due to the denaturation of the enzymes in long-time keeping in semidry conditions and the removing of the nanocomposite from the electrode surface due to its partial water-solubility in the case of PEI. The stability of the sensors and their sensitivity can be attributed to the excellent biocompatibility of the CNTs for preserving the activity of HRP and to the strong covalent interaction between CNTs, conductive polymers (polypyrrole) and the enzyme.

## 4. Conclusions

The performance of the modified glassy carbon and graphite-based screen printed electrodes as new tools for the detection and dosage of acetaminophen has been demonstrated. The immobilization of CNTs and enzyme at the surface of the electrode allows the analysis of acetaminophen with good sensitivity and selectivity. The limit of detection was in the range 1.36–8.09 μM and the linear ranges obtained for acetaminophen were between 4.08 and 79.01 μM, and 24.27 and 118.06 μM, respectively, depending on the electrode type. Real samples analysis showed a good recovery and a RSD between 1.67% and 3.27%. The recovery data obtained with the electrochemical method were in agreement with the results obtained according to the Romanian Pharmacopoeia. The strategies used offer interesting prospects for the development of other biosensors for pharmaceutical analysis, taking in consideration the simplicity of the surface modification of the electrode.

## Figures and Tables

**Table 1.** Carbon nanotube features.

**Tested CNTs**	**Length** **(μm)**	**Diameter** **(nm)**
SWCNT	1–5	∼1.5
MWCNT (type 1)	30±10	1–5
MWCNT (type 2)	30±15	1–5
MWCNT (type 3)	30±10	5–20
MWCNT (type 4)	30±15	5–20

**Table 2.** Amperometric parameters for acetaminophen determination.

**Electrode Type**	**Linear Range** **(μM)**	**Linear Regression Equation** **Y (μA) = A + BX (μM)**		**R^2^**	**LOD (μM)**

		**A**	**B**		
0.3 mg·mL^−1^ HRP/ SWCNT/PEI at GCE (Φ 1mm)	18.18–79.01	−0.003	−6.108 x10-4	0.978	6.061 ± 0.002
0.6 mg·mL^−1^ HRP/ SWCNT/PEI at GCE (Φ 1mm)	4.08–79.01	−0.023	−0.002	0.951	1.365 ± 0.013
0.3 mg·mL^−1^ HRP/ SWCNT/PEI at GCE(Φ 4mm)	23.46–79.01	−0.158	−0.020	0.965	7.823 ± 0.099
0.6 mg·mL^−1^ HRP/ SWCNT/PEI at GCE(Φ 4mm)	6.45–79.01	−0.186	−0.008	0.913	2.158 ± 0.068
0.48 mg·mL^−1^ HRP/SWCNT/Ppy at SPE	24.27–118.06	−0.070	−0.007	0.938	8.096 ± 0.102

**Table 3.** Acetaminophen electrochemical determination from monocomponent commercial pharmaceuticals.

**Electrode Type**	**Commercial Formulations**	**Added** **(μM)**	**Found** **(μM)**	**Recovery** **(%)**	**RSD** **(%)**
0.3 mg·mL^−1^ HRP/SWCNT/PEI at GCE(Φ 4mm)	Acetaminophen	10	10.01	100.10	0.98
		20	20.08	100.40	1.06

	Acetaminophen	-	10.04	100.40	1.67
	Panadol^®^	10	20.88	104.40	1.31
	GlaxoSmithKline	20	31.09	103.64	2.04

	Acetaminophen	-	9.94	99.44	2.35
	Sanador^®^	10	19.77	98.85	2.54
	Laropharm	20	28.97	96.60	2.09

0.3 mg·mL^−1^ HRP/SWCNT/PEI at graphite SPE	Acetaminophen	10	9.98	99.80	1.42
		20	20.03	100.15	1.86

	Acetaminophen Panadol^®^	-	9.91	99.10	2.81
	GlaxoSmithKline	10	20.53	102.65	2.93

**Table 4.** Determination of acetaminophen by spectrophotometry.

**Active Compound**	**Absorbance (λ = 243nm)**		**Recovery** **(%)**	**RSD** **(%)**

	**Theoretical**	**Found**		
Acetaminophen	0.645	0.6496	100.71	0.059
	
Acetaminophen Panadol^®^		0.6555	101.62	0.114
GlaxoSmithKline				
	
Acetaminophen		0.6074	94.17	0.187
PanadolExtra^®^				
GlaxoSmithKline				
